# An Assessment of Iterative Reconstruction Methods for Sparse Ultrasound Imaging

**DOI:** 10.3390/s17030533

**Published:** 2017-03-08

**Authors:** Solivan A. Valente, Marcelo V. W. Zibetti, Daniel R. Pipa, Joaquim M. Maia, Fabio K. Schneider

**Affiliations:** Graduate Program in Electrical and Computer Engineering (CPGEI), Federal University of Technology, Paraná (UTFPR), Curitiba PR 80230-901, Brazil; marcelozibetti@utfpr.edu.br (M.V.W.Z.); danielpipa@utfpr.edu.br (D.R.P.); joaquim@utfpr.edu.br (J.M.M.); fabioks@utfpr.edu.br (F.K.S.)

**Keywords:** ultrasonic imaging, image reconstruction, iterative methods

## Abstract

Ultrasonic image reconstruction using inverse problems has recently appeared as an alternative to enhance ultrasound imaging over beamforming methods. This approach depends on the accuracy of the acquisition model used to represent transducers, reflectivity, and medium physics. Iterative methods, well known in general sparse signal reconstruction, are also suited for imaging. In this paper, a discrete acquisition model is assessed by solving a linear system of equations by an $\ell_{1}$-regularized least-squares minimization, where the solution sparsity may be adjusted as desired. The paper surveys 11 variants of four well-known algorithms for sparse reconstruction, and assesses their optimization parameters with the goal of finding the best approach for iterative ultrasound imaging. The strategy for the model evaluation consists of using two distinct datasets. We first generate data from a synthetic phantom that mimics real targets inside a professional ultrasound phantom device. This dataset is contaminated with Gaussian noise with an estimated SNR, and all methods are assessed by their resulting images and performances. The model and methods are then assessed with real data collected by a research ultrasound platform when scanning the same phantom device, and results are compared with beamforming. A distinct real dataset is finally used to further validate the proposed modeling. Although high computational effort is required by iterative methods, results show that the discrete model may lead to images closer to ground-truth than traditional beamforming. However, computing capabilities of current platforms need to evolve before frame rates currently delivered by ultrasound equipments are achievable.

## 1. Introduction

The reconstruction of ultrasonic images, usually known as Mode ”B”, for ”bright”, is traditionally done by a technique called *beamforming* [1], which consists of estimating the acoustic response of a *region of interest* (ROI) after receiving ultrasound signals from a sensors array. While beamforming is widely used and constantly improved, it is mainly based on treating the received signals with time delays, demodulation, gain control and appropriate compression, without considering more sophisticated mathematical models for the transducer elements or for the ultrasound wave propagation in the imaged medium.

Exploiting such models may lead to better image reconstructions, by taking advantage of components ignored in traditional beamforming processing. The quality enhancement made possible by this approach has been shown, for example, in [2,3,4], while [5] shows that significant improvement of axial and lateral resolutions with respect to beamforming may be achieved.

The use of acquisition models for image reconstruction has been a rich field of research. The application of this concept ranges, for example, from Super-resolution [6,7,8], to Computed Tomography (CT) [9,10,11], Photoacoustic Tomography (PAT) [12,13,14], and Magnetic Resonance Imaging (MRI) [15,16], just to mention a few.

However, better images come at a cost because Inverse Problems-Based (IPB) iterative methods usually require large computational effort. Two aspects are relevant in this approach. First, a reasonable acquisition model and accurate priors are crucial to a good reconstruction. Second, fast algorithms are important to reduce the overall reconstruction time.

This paper focus on both aspects. It assesses a discrete acquisition model, formulated to represent the way an array of ultrasonic transducers emit and receive signals, and the way ultrasound waves travel in the imaged medium. Eleven well-known sparse iterative methods are presented, their algorithms are described and their optimization parameters are examined, with the goal of finding the best approach for ultrasound imaging. These methods have been chosen because they have shown to be very effective in many signal processing applications, including image reconstruction. See [17] and the following references in each method for details.

In the IPB approach, the desired image can be estimated by solving a discrete mapping, which is often written in the form of a linear system of equations g=Hf, where the vector of unknowns $f\in R^{m}$ represents the desired image, the matrix $H\in R^{n\times m}$ represents the discrete acquisition model, and the data read from a sensors array are stored in vector $g\in R^{n}$ [18].

Given some assumptions about the data and the signal statistical behaviour (e.g., sparsity), it is possible to find an approximate solution to the system of equations by minimizing a $\ell_{1}$-norm regularized least squares problem. Referred henceforth as an $\ell_{2}$–$\ell_{1}$ problem, it consists of a squared $\ell_{2}$-norm term plus an $\ell_{1}$-norm term, usually weighted by a factor *λ*. This sparse estimation problem is usually posed as:

(1) $$\hat{f}=\arg\min_{f}\Psi \left(f\right)=\arg\min_{f}\frac{1}{2}{∥g-\mathrm{Hf}∥}_{2}^{2}+\lambda {∥f∥}_{1}.$$

Solving (1) implies a pointwise sparsity prior assumption for f, which is assumed to be true in this study. If a regionwise sparsity is to be assumed, one may solve for f by minimizing with a modified regularization term:

$$\hat{f}=\arg\min_{f}\bar{\Psi }\left(f\right)=\arg\min_{f}\frac{1}{2}{∥g-\mathrm{Hf}∥}_{2}^{2}+\lambda R\left(f\right)$$

where *R* may be the *Total Variation* (TV) operator, R(f)=TV(f) or any other appropriate linear transformation, such as $R\left(f\right)={∥\mathrm{Lf}∥}_{1}$ or $R\left(f\right)={∥L\;|f|\;∥}_{1}$ (e.g., see [19,20]), where L implements Finite Differences, Wavelet or another convenient sparsifying transform.

The model represented by matrix H plays a key role in the reconstruction. In this paper, its evaluation strategy consists of assessing the images yielded by two distinct datasets. We first generate data from a synthetic phantom image, where f is known and mimics the real targets inside a professional ultrasound phantom device. These data are then contaminated with Gaussian noise, whose SNR is estimated from real data. This ’synthetic’ case evaluation is necessary in order to compare the reconstructed images against a previously known result, using a proper ultrasound imaging metric. Second, we evaluate the discrete model with real data collected by a research ultrasound platform when scanning the same phantom device. In order to further validate the proposed modeling, a distinct real dataset is finally used with its proper matrix H and results are also compared with the corresponding beamforming image.

The chosen iterative methods are known to be suitable to minimize $\ell_{2}$–$\ell_{1}$ cost functions such as Ψ(f). They present different strategies for convex optimization, and are divided here in four groups for convenience: (i) four variants from the *Fast Iterative Shrinkage-Thresholding Algorithm* (FISTA) family [21,22]; (ii) four variants of the *Iterative Re-weighted Least Squares* (IRLS) method [23]; (iii) two variants of the *Nonlinear Conjugate Gradient* (NLCG) method [24,25], and (iv) an Augmented Lagrangian-based algorithm called *Alternating Direction Method of Multipliers* (ADMM) [26,27].

In addition to parameter *λ* in Equation (1), some of these algorithms have other parameters to be set in order to achieve their optimal results for a particular application. In this study, the parameters of all methods are examined and tuned independently and only the best results are shown.

## 2. The Ultrasonic Acquisition Model

The following linear model utilized for building the acquisition matrix H is based on the work of ultrasound community researchers, in [28,29,30,31] and [3,32,33,34,35,36,37,38]. This kind of modeling has been investigated for general ultrasound imaging, as in [3,39], as well as for medical ultrasound imaging, as in [2,40], in order to allow image reconstruction by the IPB approach, as proposed in this study.

In the experiments presented in this paper, matrix H was built using *Field II* software package [41,42], running on MATLAB^®^ (The MathWorks Inc., Natick, MA, USA) environment.

### 2.1. Ultrasonic Pulse Transmission

Let $u_{k}\left(t\right)$ be the electric pulse applied to the *k*-th transducer element, $h_{k}^{ef}\left(t\right)$ be its *forward* electro-acoustic impulse response, and $h_{k}^{f-SIR}\left(r,t\right)$ be the *forward* spatial impulse response that models the physical medium between the emitter and a point r at the imaged region, at time *t*. The *acoustic pressure* $p_{k}\left(r,t\right)$ at point r associated to the signal coming from this element is given by:

(2) $$p_{k}\left(r,t\right)=h_{k}^{f-SIR}\left(r,t\right)*h_{k}^{ef}\left(t\right)*u_{k}\left(t\right),$$

where ∗ denotes temporal convolution.

As in many applications, we consider the electro-acoustic impulse response to be modeled as a cosine modulated Gaussian pulse [5]:

(3) $$h^{ef}\left(t\right)=e^{{\left(-\alpha .BW.t\right)}^{2}}\cos\left(2\pi f_{c}t\right)$$

where BW stands for the pulse *fractional bandwidth* (the ratio between the transducer bandwidth and its central frequency $f_{c}$), and *α* is an attenuation factor. In this study, we consider all transducers elements as sharing the same forward electro-acoustic response.

The forward spatial impulse response is given by:

(4) $$h_{k}^{f-SIR}\left(r,t\right)=\int_{S}\frac{\delta (t-\frac{\left|r\right|}{v})}{2\pi |r|}dS,$$

where $\delta (t-\frac{\left|r\right|}{v})$ is a shifted Dirac’s delta function, that allows the contribution of the *k*-th element along the area *S* to be added only when the point r is stimulated by the corresponding ultrasound wave, which travels at a constant speed *v*. This spatial impulse response models the acoustic field decreasing as the wave travels through a linear homogeneous *non-attenuating* medium [43]. When considering attenuation, Equation (4) is modified to become:

(5) $$h_{k}^{f-SIR}\left(r,t\right)=\int_{T}\int_{S}a\left(t-\tau ,|r|\right)\frac{\delta (\tau -\frac{\left|r\right|}{v})}{2\pi |r|}dSd\tau ,$$

where *a* is the *attenuation impulse response*, and attenuation is assumed to be the same throughout the medium [32]. In this study, *Field II* software package is set to consider both frequency-independent and frequency-dependent attenuations. As the model is intended to represent a specific propagation medium, the attenuation rates are obtained from the professional phantom scanned to collect the real dataset. Details are given in Section 4.

Assuming the propagation medium has linear behaviour, the contribution of all *K* transducer elements for the acoustic pressure at point r over time is given by summing all $p_{k}\left(r,t\right)$:

(6) $$p\left(r,t\right)=\sum_{k=1}^{K}h_{k}^{f-SIR}\left(r,t\right)*h_{k}^{ef}\left(t\right)*u_{k}\left(t\right).$$

### 2.2. Ultrasonic Pulse Reflection (Echo)

Each point of the imaged region has a distinct response, varying according to the acoustic impedance of this particular portion of the imaged object. In fact, impedance *differences* in the propagation medium are responsible for partial or total reflection of the incident waves, and they essentially occur due to varying densities, different types of materials, or due to boundaries between structures or organs.

The amount of reflection at position r is quantified by f(r), where 0 indicates no reflection and -1 or +1 full reflection, with or without phase inversion, respectively. This quantity is also known as *amplitude reflection coefficient*, and differs from the *intensity reflection coefficient*, which relates the incident and reflected *energies* at an observation point [36].

Point r may thus be considered an acoustic emitter, where waves have initial amplitude given by p(r,t)f(r), and travel back to all *K* transducer elements.

Let $h_{n}^{b-SIR}\left(r,t\right)$ be the *backward* spatial impulse response, which models the medium between the emitter at r and the *n*-th transducer element at time *t*. Let also $h_{n}^{eb}\left(t\right)$ be the backward or *reverse* electro-acoustic impulse response of such *n*-th element. According to [5], it is reasonable to consider all transducers elements as sharing the same forward and the same reverse electro-acoustic impulse responses. It is also plausible to assume the forward and backward spatial impulse responses to be equal, as the medium is supposed linear. Therefore, both assumptions are adopted in this study.

We may then estimate the electric signal $g_{n}\left(r,t\right)$ generated by this receiving element over time, associated with a particular point r:

(7) $$g_{n}\left(r,t\right)=h_{n}^{eb}\left(t\right)*h_{n}^{b-SIR}\left(r,t\right)*p\left(r,t\right)f\left(r\right).$$

Combining Equations (6) and (7) we obtain the overall impulse response for the *n*-th receiving transducer element, $h_{n}\left(r,t\right)$:

(8) $$h_{n}\left(r,t\right)=h_{n}^{eb}\left(t\right)*h_{n}^{b-SIR}\left(r,t\right)*\left[\sum_{k=1}^{K}h_{k}^{f-SIR}\left(r,t\right)*h_{k}^{ef}\left(t\right)*u_{k}\left(t\right)\right],$$

and Equation (7) becomes:

(9) $$g_{n}\left(r,t\right)=h_{n}\left(r,t\right)f\left(r\right).$$

Integrating and discretizing Equation (9) over the ROI, we get:

(10) $$g_{n}\left[t_{i}\right]=\sum_{r\in ROI}h_{n}\left[r,t_{i}\right]f\left[r\right]+e_{n}\left[t_{i}\right]$$

where $t_{i}$ are the sampling time points, r∈ROI are points in space, and $e_{n}$ accounts for the discretization errors and for the noise present in $g_{n}$.

The implicit errors in this sampling procedure are well-known in general signal processing literature and in the image science context (e.g., [18,44]). In this study, we have used a sampling frequency of four times the ultrasonic pulse frequency for time discretization, and a one wavelength resolution for the ROI discretization. While the Nyquist criterion guarantees no data loss in time sampling, we do miss spatial information from the interstices, i.e., from the intervening spaces between the mapped points.

### 2.3. The System Model

We can arrange Equation (10) in a matrix form, with one expression for each transducer element, by writing $g_{n}=H_{n}f+e_{n}$. Or, more conveniently, we can combine all *K* transducer elements into one single matrix equation, stacking vectors and matrices appropriately:

$$\left[\begin{array}{c} g_{1}\\ g_{2}\\ \vdots \\ g_{K} \end{array}\right]=\left[\begin{array}{c} H_{1}\\ H_{2}\\ \vdots \\ H_{K} \end{array}\right]f+\left[\begin{array}{c} e_{1}\\ e_{2}\\ \vdots \\ e_{K} \end{array}\right]$$

(11) g=Hf+e,

where the linear system represents the acquisition process.

The structure of matrix H depends on the choice for the positions of the array elements in the coordinate system used for locating points r in the ROI. In this study, we have adopted a symmetric distribution, with the transducers array placed parallel to the superior border of the ROI. Half the elements are at the left side of the ROI centre axe, and half at the right side.

**Figure 1:**
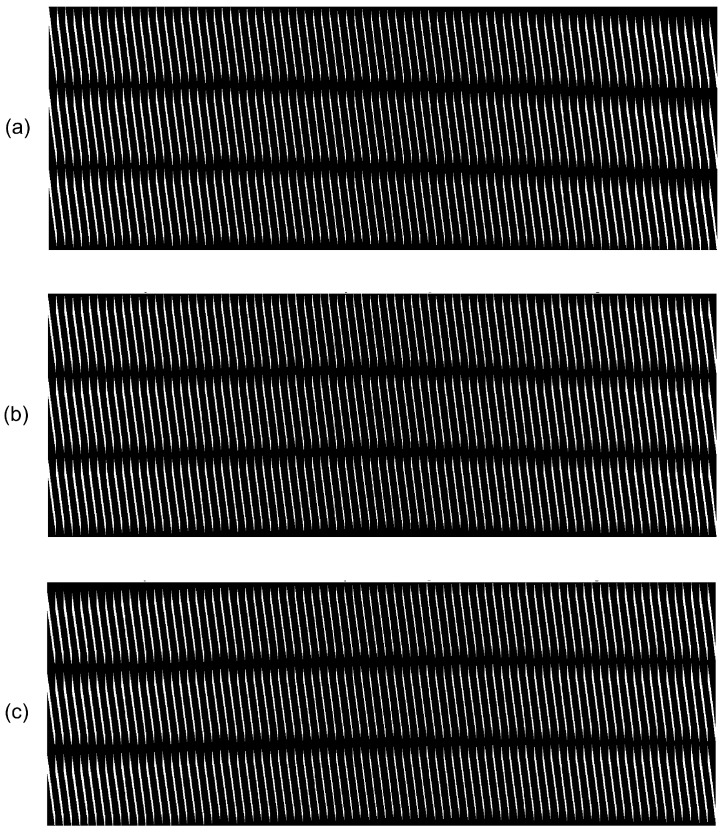
Depiction of the model matrix H. (**a**) On top, three stripes rows represent the sub-matrices $H_{1}$ to $H_{3}$; (**b**) In the middle, the sub-matrices $H_{31}$ to $H_{33}$; (**c**) In the bottom, the sub-matrices $H_{62}$ to $H_{64}$.

As detailed later, this study uses K=64 array elements. Figure 1 shows the internal structure of matrix H. The black regions in the picture indicate null elements, while points in white represent non-null elements; it is clear that H is somewhat sparse.

Recalling the 2D image is represented by the 1D vector $f\in R^{m}$, the matrix $H\in R^{n\times m}$ has as many columns as there are pixels in the image. As the 2D image columns are stacked in f, this explains the periodic diagonal stripes standard in each sub-matrix $H_{n}$.

**Figure 2:**
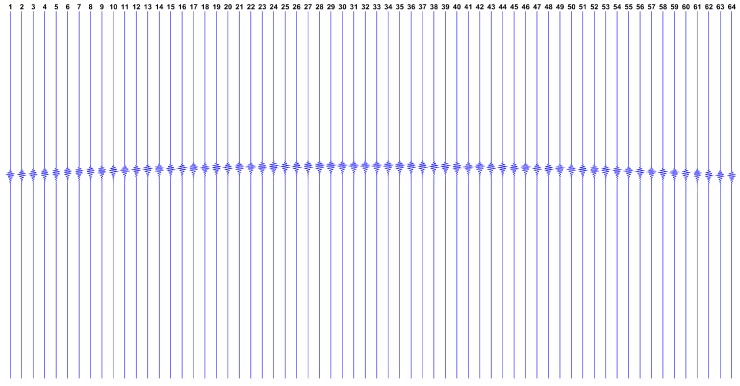
Expected dataset g from the transducers array, with a single reflector phantom at the region of interest (ROI) center. Each vertical line represents the dataset $g_{n}$ from a single transducer, over time $t_{i}$ (increasing from top to bottom).

**Figure 3:**
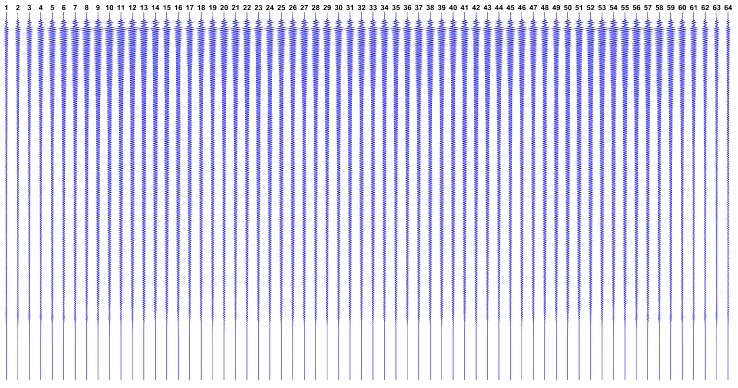
Expected dataset g from the transducers array, with reflectors at every ROI pixel. Notice the effect of the attenuation over time $t_{i}$, showing that lower signal levels are expected from distant ROI points.

Now, observing the expected datasets issued by this model, Figure 2 shows how g looks like if we have a single reflector at the ROI center, while Figure 3 shows g in the hypothetical case where all ROI points have reflectors.

When using this symmetrical positioning of transducers in relation to the ROI, it is possible to simplify the computation of H by only performing the calculations for one side of the ROI (e.g., the left), and mirroring the elements of H that correspond to the other side. This strategy halves the computation time for building the model.

Another possibility for building the acquisition model is to avoid computing and storing the whole matrix H, given we know how to calculate each of its elements. As the size of images to be reconstructed increases, the dimensions of H grow very rapidly. This may considerably slow down the reconstruction process and may even render it prohibitive, given the memory limitation of current computing platforms. To work around this problem, we may compute elements of H as the reconstruction process takes place, given that parallel processing platforms may easily handle this task, avoiding the use of expensive large amounts of memory.

## 3. Review of the Algorithms

For notation simplicity, from this point on we shall denote the linear system of equations by g=Hf, letting the error vector e to be implicit inside the dataset g, unless stated otherwise.

A naive solution to the optimization problem would be utilizing matrix inversion to get $\hat{f}=H^{-1}g$, but this rarely leads to a meaningful solution, because H is usually ill-conditioned in many imaging applications. The problem of estimating the vector f is known as an *inverse problem*, and the estimation itself is sometimes called reconstruction or *deconvolution*. [18]

The solution to an inverse problem is usually formulated as a minimization of some functional. Part of this functional measures the discrepancy between the measured data g and the model-generated signal Hf. The first term of Ψ(f) in Equation (1) represents this discrepancy and, assuming a Gaussian statistical behaviour for the residue g-Hf, we use the squared $\ell_{2}$-norm as an optimal discrepancy measurement in the maximum likelihood sense. [45]

The other part of the functional is usually a *prior* or *regularization* term, which is necessary to stabilize the ill-posed problem. The second term of Ψ(f) uses the $\ell_{1}$-norm, which comes from a Laplace distribution on a Bayesian sense [46], and promotes a sparse solution f. However, this prior brings an inconvenience, since solving an $\ell_{1}$-norm regularized least squares corresponds to a *non-linear* system of equations, that needs to be solved iteratively. Up to now, there is no method elected as the best one for $\ell_{2}$–$\ell_{1}$ problems, specially for ultrasound reconstruction. In this study, we evaluate some methods suitable for this kind of problem, which are reviewed below.

We note that the study of algorithms for $\ell_{2}$–$\ell_{1}$ optimization is a very active field of research, and this paper is not intended to bring the reader the utmost achievements in the area. Some recent results on the topic may be found, for instance, in [20,47,48,49,50] and in the references therein. Ultrasonic-specific methods have also been developed, as e.g., in [51].

### 3.1. General and $\ell_{2}$–$\ell_{1}$ Specific Line Search

Iterative methods for convex optimization are usually based on some form of gradient, and their performances may greatly depend on how much the algorithm advances in a given search direction. The amount of progress toward the minimum is adjusted by the so-called *stepsize* parameter. In some algorithms the stepsize is fixed, while in others its optimal value is computed at each iteration. In these cases, an optimization procedure takes place at each iteration, but it usually represents a small computation overload as the problem dimensions increase.

Usually called *line search*, the stepsize calculation is performed as a minimization along the line defined by the search direction. Given a current solution $f_{k}$ and a search direction $d_{k}$ at the *k*-th iteration, the line search procedure consists of an unidimensional minimization, once we evaluate the cost function only regarding the stepsize *α* as:

(12) $$\alpha_{k}=\arg\min_{\alpha }\Psi \left(f_{k}+\alpha d_{k}\right).$$

Although many general line search methods may apply to $\ell_{2}$–$\ell_{1}$ minimization, we may not be certain to reach the minimum in a reasonable time. This is why some researchers have recently proposed specific $\ell_{2}$–$\ell_{1}$ line search procedures, that have shown to be more efficient than more general methods. See for instance [10,52,53] and the references therein.

Some methods assessed in this paper use a line search procedure. They are all identified with an ”OLS” suffix, standing for *Optimal Line Search*. For unity, we have adopted in all cases the procedure proposed in [53].

### 3.2. FISTA

Fast Iterative Shrinkage-Thresholding Algorithms (FISTA) are named after the shrinkage-thresholding operator $S_{a}\left(x\right)$, defined as:

(13) $$S_{a}\left(x\right)=\left\{\begin{array}{cc} 0 & ,\;a\geq |x|\\ x-a\;\;\mathrm{sign}(x) & ,a<|x| \end{array}\right..$$

Relatively recent, the methods belonging to this family have shown to be very efficient for $\ell_{2}$–$\ell_{1}$ minimization, especially with high-dimensional problems. They all rely on the same basic steps, namely the calculation of the residual vector g-Hf (in the data space), its back projection to the image space by multiplication by $H^{T}$, followed by a shrinkage-thresholding step [17]. Iterations proceed until some stop criterion is reached. FISTA was proposed in [21] as an accelerated alternative to the ISTA family of methods [54], providing a convergence order of $1/k^{2}$, instead of 1/k, where *k* is the iteration index.

As seen in Algorithm 1, at each iteration the algorithm estimates the new $f_{k}$ via the shrinkage-thresholding operator, applied to each element of a resulting vector, obtained by summing a special point $y_{k}$ to the negative of the gradient at this point.

Point $y_{k}$ is calculated by combining two previous solutions, and it represents the key modification that accelerates FISTA with respect to previous ISTA methods. As iterations evolve, the vector $y_{k}$ modifies the solution point over which the sparsity promoting shrinkage-thresholding operator is applied, gradually improving an algorithmic feature know as *momentum*, providing good convergence speed. While fast, FISTA requires a few iterations to achieve its acceleration, since it needs to gain momentum while iterates. This means its first iterations are very similar to the ones of a non-accelerated ISTA method.

**Algorithm 1** Fast Iterative Shrinkage-Thresholding Algorithm (FISTA) [21].**Require:**
*λ*, $c\geq {∥H^{T}H∥}_{2}$
1:**set** $f_{0}=0$2:**set** $y_{1}=f_{0}$3:**set** $t_{1}=1$4:**set** k=15:**while** *stop criterion not reached* **do**6:  **set** $f_{k}=S_{\lambda /c}\left[\frac{1}{c}H^{T}\left(g-{\mathrm{Hy}}_{k}\right)+y_{k}\right]$7:  **set** k=k+18:  **set** $t_{k}=\left(1+\sqrt{1+4t_{k-1}^{2}}\right)/\;2$9:  **set** $y_{k}=f_{k-1}+\frac{t_{k-1}-1}{t_{k}}\left(f_{k-1}-f_{k-2}\right)$10:**end** **while**


FISTA convergence is guaranteed for values of the parameter *c* greater than the Lipschitz constant of the gradient of the cost function differentiable term, that is, the squared $\ell_{2}$-term. This constant is related to greatest singular value of matrix $H^{T}H$; a typical condition is to set $c\geq {∥H^{T}H∥}_{2}$. [21]

While faster than other methods from its family, FISTA is not a monotonic algorithm because it does not guarantee a new solution $f_{k}$ to be lower in the cost function than the previous one. Therefore, the same researchers have proposed the *Monotone Fast Iterative Shrinkage-Thresholding Algorithm* (MFISTA) shortly thereafter [19]. MFISTA algorithm is shown in Algorithm 2. Monotonicity is achieved in Step 7, using a new intermediate point $z_{k}$, that also appears in a new form for the special point $y_{k}$.

**Algorithm 2** Monotone Fast Iterative Shrinkage-Thresholding Algorithm (MFISTA) [19].**Require:**
*λ*, $c\geq {∥H^{T}H∥}_{2}$
1:**set** $f_{0}=0$2:**set** $y_{1}=f_{0}$3:**set** $t_{1}=1$
4:**set** k=1
5:**while** *stop criterion not reached* **do**
6:  **set** $z_{k}=S_{\lambda /c}\left[\frac{1}{c}H^{T}\left(g-{\mathrm{Hy}}_{k}\right)+y_{k}\right]$7:  **set** $f_{k}=\arg\min_{f}\Psi \left(f\right)\;|\;f\in \left\{z_{k},f_{k-1}\right\}$8:  **set** k=k+19:  **set** $t_{k}=\left(1+\sqrt{1+4t_{k-1}^{2}}\right)/\;2$10:  **set** $y_{k}=f_{k-1}+\frac{t_{k-1}-1}{t_{k}}\left(f_{k-1}-f_{k-2}\right)\;\;\;+\frac{t_{k-1}}{t_{k}}\left(z_{k-1}-f_{k-1}\right)$11:**end** **while**



The *Over-Relaxation of Monotone Fast Iterative Shrinkage-Thresholding Algorithm* (we designate as OMFISTA) was proposed in [22]. This variant is shown in Algorithm 3, and calculates the new estimated solution $f_{k}$ using a variable stepsize $\alpha_{k}$. While using the same intermediate point $z_{k}$ from MFISTA, the new estimated solution $f_{k}$ is now obtained by comparing the cost function value from the previous iteration with the one achieved by advancing with the given stepsize in a special direction.

**Algorithm 3** Over-Relaxation of Monotone Fast Iterative Shrinkage-Thresholding Algorithm (OMFISTA) [22].**Require:**
$\alpha_{1},\eta_{1},\lambda$, $c\geq {∥H^{T}H∥}_{2}$
1:**set** $f_{0}=0$
2:**set** $y_{1}=f_{0}$
3:**set** $t_{1}=\alpha_{1}$4:**set** k=15:**while** *stop criterion not reached* **do**
6:  **set** $z_{k}=S_{\lambda /c}\left[\frac{1}{c}H^{T}\left(g-{\mathrm{Hy}}_{k}\right)+y_{k}\right]$7:  **calculate** $\alpha_{k}$ *(via line search if OLS)*8:  **set** $f_{k}=\arg\min\Psi \left(f\right)|\;\;f\in \{f_{k-1}+\alpha_{k}\left(z_{k}-f_{k-1}\right),f_{k-1}\}$9:  **set** k=k+110:  **set** $t_{k}=\left(\alpha_{1}\alpha_{k-1}+\sqrt{\alpha_{1}^{2}\alpha_{k-1}^{2}+4t_{k-1}^{2}}\right)/\;2$
11:  **set** $y_{k}=f_{k-1}+\frac{t_{k-1}-\alpha_{1}}{t_{k}}\left(f_{k-1}-f_{k-2}\right)\;\;\;+\frac{t_{k-1}}{t_{k}}\left(z_{k-1}-f_{k-1}\right)\;\;\;+\frac{t_{k-1}}{t_{k}}\left(1-\eta_{k-1}\right)\left(y_{k-1}-z_{k-1}\right)$12:**end** **while**



This formulation allows the algorithm to be tuned with a properly chosen stepsize, that may vary or be fixed as iterations evolve. At the cost of having this additional parameter to be set, OMFISTA may achieve faster performance than MFISTA. As $t_{k}$, the point $y_{k}$ also takes a different form. The new parameter $\eta_{k}$ depends on the chosen *c* and on the stepsize $\alpha_{k}$, among other factors [22]. In results presented in this study, we have considered $\alpha_{1}$ and $\alpha_{k}$ as a single constant parameter *α*, and $\eta_{k}$ as a constant *η*.

Finally, we assess the *Over-Relaxation of Monotone Fast Iterative Shrinkage-Thresholding Algorithm with Optimal Line Search* (OMFISTA-OLS) as a variant of OMFISTA proposed in [10] for computed tomography. Here, the stepsize $\alpha_{k}$ is calculated by the line search procedure proposed in [53], as opposed to the fixed stepsize used in OMFISTA.

### 3.3. NLCG

The linear *Conjugate Gradient* (CG) method is one of the main algorithms to solve large dimension linear systems of equations. It is particularly interesting for ill-conditioned matrices because it converges quicker than methods based only on gradient, and has guaranteed convergence in a finite number of iterations. [24,55]

Derived from CG, the *Nonlinear Conjugate Gradient* (NLCG) method is proper for solving nonlinear systems, such as Equation (1). While similar, NLCG differs from CG in some aspects. One of them is the parameter *β*, a key element to define the search direction at a given iteration. Considering a system with a coefficients matrix A, this parameter ensures the next search direction is always A-orthogonal to all previous ones in the linear case, promoting convergence in a finite amount of steps. However, in the nonlinear case this is no longer true [24], and many researchers have proposed different choices for *β*. An interesting survey on these many proposals is presented in [25].

The NLCG algorithm is presented in Algorithm 4, where r stands for the residual, W is a diagonal weight matrix that uses a small δ>0 parameter to approximate the $\ell_{1}$-norm for a strictly convex surrogate, *α* is the stepsize in the search direction d, and y is an auxiliary variable. The choice for *β* expressed in step 14 was proposed by Hestenes and Stiefel [56], as the *Parameters evaluation* section summarizes.

We also assess a variant of NLCG we designate *Nonlinear Conjugate Gradient with Optimal Line Search* (NLCG-OLS). It is obtained by replacing the standard stepsize from step 8 with the one calculated by the aforementioned $\ell_{2}$–$\ell_{1}$ optimal line search from [53]. This iteration-dependent stepsize calculation allows the algorithm to converge faster to the minimum.

**Algorithm 4** Nonlinear Conjugate Gradient (NLCG) [24,55].**Require:**
δ,λ
1:**set** $f_{1}=0$
2:**set** $r_{1}=g-{\mathrm{Hf}}_{1}$
3:**set** $W_{1}=diag\left[1/(|f_{1}|+\delta )\right]$
4:**set** $\nabla \Psi_{1}=-H^{T}\left(g-{\mathrm{Hf}}_{1}\right)+\lambda W_{1}f_{1}$
5:**set** $d_{1}=-\nabla \Psi_{1}$
6:**set** k=1
7:**while** *stop criterion not reached* **do**
8:  **set** $\alpha_{k}=\frac{-\nabla \Psi_{k}^{T}d_{k}}{\left[{\left({\mathrm{Hd}}_{k}\right)}^{T}\left({\mathrm{Hd}}_{k}\right)+\lambda \left(d_{k}^{T}W_{k}d_{k}\right)\right]}$9:  **set** $f_{k+1}=f_{k}+\alpha_{k}d_{k}$10:  **set** $r_{k+1}=r_{k}-\alpha_{k}{\mathrm{Hd}}_{k}$
11:  **set** $W_{k+1}=diag\left[1/(|f_{k+1}|+\delta )\right]$12:  **set** $\nabla \Psi_{k+1}=-H^{T}r_{k+1}+\lambda W_{k+1}f_{k+1}$13:  **set** $y_{k}=\nabla \Psi_{k+1}-\nabla \Psi_{k}$14:  **set** $\beta_{k}=\left(\nabla \Psi_{k+1}^{T}y_{k}\right)/\left(d_{k}^{T}y_{k}\right)$
15:  **set** $d_{k+1}=-\nabla \Psi_{k+1}+\beta_{k}d_{k}$16:  **set** k=k+117:**end** **while**



### 3.4. IRLS

The *Iteratively Re-weighted Least Squares* (IRLS) method is well known in the literature [23] and it is a simple and attractive option for solving nonlinear systems of equations such as Equation (1). It consists of obtaining a re-weighted quadratic approximation of the cost function at each iteration, and then calculating its least squares solution. The IRLS method is shown in Algorithm 5.

**Algorithm 5** Iteratively Re-weighted Least Squares (IRLS) [23].**Require:**
δ,λ
1:**set** $f_{1}=0$
2:**set** k=1
3:**while** *stop criterion not reached* **do**
4:  **set** $r_{k}=g-{\mathrm{Hf}}_{k}$5:  **set** $\nabla f_{k}=-H^{T}r_{k}$
6:  **set** $W_{k}=diag\left[1/(|f_{k}|+\delta )\right]$7:  **set** $d_{k}={\left(H^{T}H+\lambda W_{k}\right)}^{-1}\left(-\nabla f_{k}-\;\lambda W_{k}f_{k}\right)$8:  **set** $f_{k+1}=f_{k}+d_{k}$
9:  **set** k=k+110:**end** **while**



While IRLS clearly uses an unitary stepsize (see Step 8), a variant we call *Iteratively Re-weighted Least Squares with Optimal Line Search* (IRLS-OLS) is obtained by explicitly calculating an $\alpha_{k}$ stepsize with the line search procedure from [53]. In this case, the new solution $f_{k+1}$ becomes $f_{k}+\alpha_{k}d_{k}$ instead of $f_{k}+d_{k}$. Here again, the adaptive stepsize calculation at each iteration promotes faster convergence as the results shall illustrate.

Another assessed variant of IRLS consists of using the CG method for solving $\left(H^{T}H+\lambda W_{k}\right)d_{k}=\left(-\nabla f_{k}-\lambda W_{k}f_{k}\right)$ for $d_{k}$, instead of explicitly calculating the inverse ${\left(H^{T}H+\lambda W_{k}\right)}^{-1}$, as in Step 7. We designate this variant as the *Iteratively Re-weighted Least Squares with Conjugate Gradient* (IRLS-CG) method. Its convergence speed advantage over the classical IRLS depends on the problem size, as the matrix inversion may be more or less time-consuming than the iterative CG solving method.

The fourth variant of IRLS is called *Iteratively Re-weighted Least Squares with Conjugate Gradient and Optimal Line Search* (IRLS-CG-OLS). It combines the use of the CG method to calculate the search direction $d_{k}$ at each iteration, and the line search procedure from [53] to set the appropriate stepsize in that direction.

### 3.5. ADMM

The *Alternating Direction Method of Multipliers* (ADMM) was initially proposed in [26] and in [27], while [57] shows its application to convex optimization and in particular to large scale problems. The method proposes to solve a convex problem with an equality constraint by using the *Method of Multipliers* with the *Augmented Lagrangian* operator.

Considering the minimization problem:

(14) $$\begin{array}{c} \min\;\;\;q(x),\\ s.t.\text{:}\;\;\;\mathrm{Ax}=b, \end{array}$$

the Lagrangian is given by:

(15) $$L\left(x,y\right)=q\left(x\right)+y^{T}\left(\mathrm{Ax}-b\right),$$

and the so-called *dual function* is:

(16) $$r\left(y\right)=\underset{x}{\inf}L\left(x,y\right)=-q^{*}\left(-A^{T}y\right)-b^{T}y,$$

where y is the *dual variable* (or Lagrange multiplier) and $q^{*}$ is the convex conjugate function of *q*. The dual problem is to maximize r(y) with respect to y. [58]

The convergence of Lagrangian based methods, however, depends on some special assumptions for the *q* function, such as finiteness and strict convexity. Convergence no longer depends on this assumptions when Equation (15) receives an additional term, yielding the Augmented Lagrangian:

(17) $$L_{\rho }\left(x,y\right)=q\left(x\right)+y^{T}\left(\mathrm{Ax}-b\right)+\frac{\rho }{2}{∥\mathrm{Ax}-b∥}_{2}^{2},$$

where ρ>0 is called *penalty parameter*. In this case, the dual function becomes:

(18) $$r_{\rho }\left(y\right)=\underset{x}{\inf}L_{\rho }\left(x,y\right)$$

The algorithm that solves Equation (14) is called Method of Multipliers and consists of the following steps, where the subscripts indicate the iteration indexes:

(19) $$x_{k+1}=\arg\min_{x}L_{\rho }\left(x,y_{k}\right)$$

(20) $$y_{k+1}=y_{k}+\rho \left({\mathrm{Ax}}_{k+1}-b\right)$$

These two steps represent, respectively, the minimization over x and the dual variable update by the evaluation of the equality constraint residual. [57]

In the case of the minimization of Equation (1), the use of ADMM consists of performing a variable split, and writing the cost function as a sum of two functions of different variables, namely x and f, related by an equality constraint:

(21) $$\begin{array}{c} \;\min\;\;\frac{1}{2}{∥g-\mathrm{Hx}∥}_{2}^{2}+\lambda {∥f∥}_{1},\\ \;s.t.\text{:}\;x-f=0. \end{array}$$

The solution is iteratively obtained in three steps, as shown in the loop of Algorithm 6. Here, $I\in R^{m\times m}$ is an identity matrix, *ρ* is the penalty parameter, and step 6 uses the same shrinkage-thresholding operator defined in Equation (13).

**Algorithm 6** Alternating Direction Method of Multipliers (ADMM) [26,27,57].**Require:**
ρ,λ
1:**set** $f_{0}=0$
2:**set** $y_{0}=0$
3:**set** k=1
4:**while** *stop criterion not reached* **do**5:  **set** $x_{k}=\;{\left(H^{T}H+\rho I\right)}^{-1}\left(H^{T}g+\rho f_{k-1}-y_{k-1}\right)$6:  **set** $f_{k}=\;S_{\lambda /\rho }\left(x_{k}+\frac{y_{k-1}}{\rho }\right)$
7:  **set** $y_{k}=\;y_{k-1}+\rho \left(x_{k}-f_{k}\right)$8:  **set** k=k+19:**end** **while**



### 3.6. Parameters Evaluation

#### 3.6.1. Sparsity Regulating Parameter *λ*

The parameter *λ* in Equation (1) plays an important role of balancing between the quadratic term $||g-{\mathrm{Hf}||}_{2}^{2}$ and the sparsity-promoting regularization term ${\left||f|\right|}_{1}$. For λ>0, the more we increase *λ*, the more the solution departs from the least squares minimum, which is usually noisy. This may yield a more convenient regularized solution, given a prior assumption of image sparsity.

A method for estimating *λ* is the so-called *L-curve*, initially proposed in [59] and further explored in [60]. This graphical method is probably the most convenient for selecting the regularizing parameter, as it consists of plotting the curve of the $\ell_{1}$-norm of the estimated solution ${\hat{f}}_{\lambda }$ for the correspondent $\ell_{2}$-norm of the residual, for many different *λ* values. However, the L-curve should only be used as a tool for an initial estimation of *λ*, because in applications such as ultrasonic imaging the end user must be considered when balancing between a more or less noisy image. In real platforms using the IPB approach for ultrasonic imaging, it would be reasonable to let *λ* be an adjustable parameter, as others already present in conventional beamforming machines.

**Figure 4:**
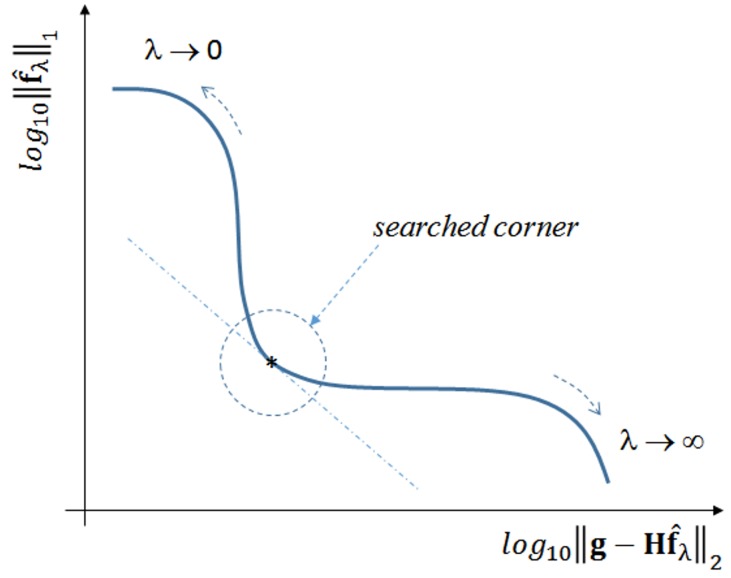
L-curve generic form. Adapted from [61].

When plotted in $log_{10}$ scale, the general expected form is an L-shaped curve, as depicted in Figure 4.

As λ→0, ${\hat{f}}_{\lambda }$ tends to the least squares solution, and the image tends to be noisy. On the other hand, the filtering effect gets more prominent as *λ* increases, forcing sparsity to the limit where $\left|\right|{\hat{f}}_{\lambda }{\left|\right|}_{1}=0$. A good compromise may be achieved by using a *λ* value that corresponds to the corner region of the curve, which is not always exactly identifiable.

Theoretically, *λ* could tend to infinity, but beyond a certain value we get $\left|\right|{\hat{f}}_{\lambda }{\left|\right|}_{1}=0$. This value is:

(22) $$\lambda_{max}=\max\left(|{\left[H^{T}g\right]}_{i}|\right),\;\mathrm{for}\;1\leq i\leq m,$$

because this is the upper limit, above which the shrinkage-thresholding operator tends to result zero (e.g., consider Step 6 in Algorithm 1 with $y_{k}=0$). In this study, the actual value used in all algorithms is set as a fraction of this upper bound by:

(23) $$\lambda =\kappa \cdot \lambda_{max},\;\;\mathrm{where}\;\;0<\kappa <1.$$

**Figure 5:**
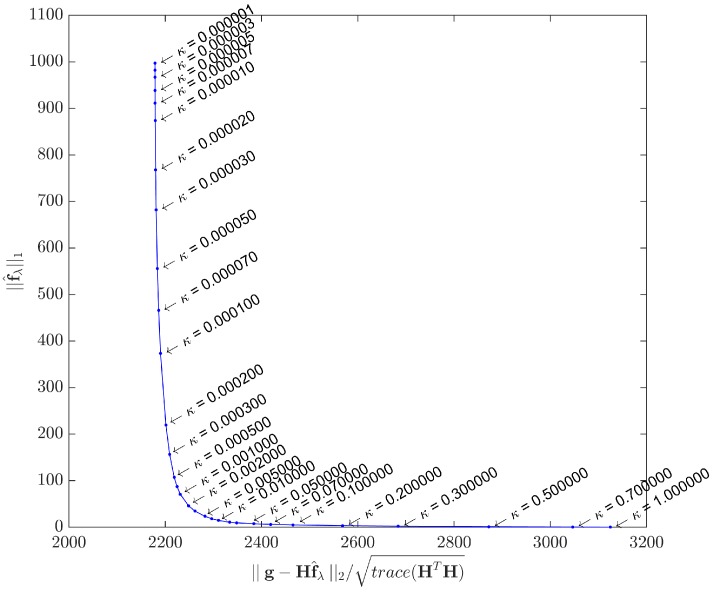
L-curve by IRLS method - Synthetic dataset. Parameter *κ* ranges from $10^{-6}$ to 1. The curvature region is approximately delimited by $\kappa =5\times 10^{-4}$ and $\kappa =5\times 10^{-2}$.

**Figure 6:**
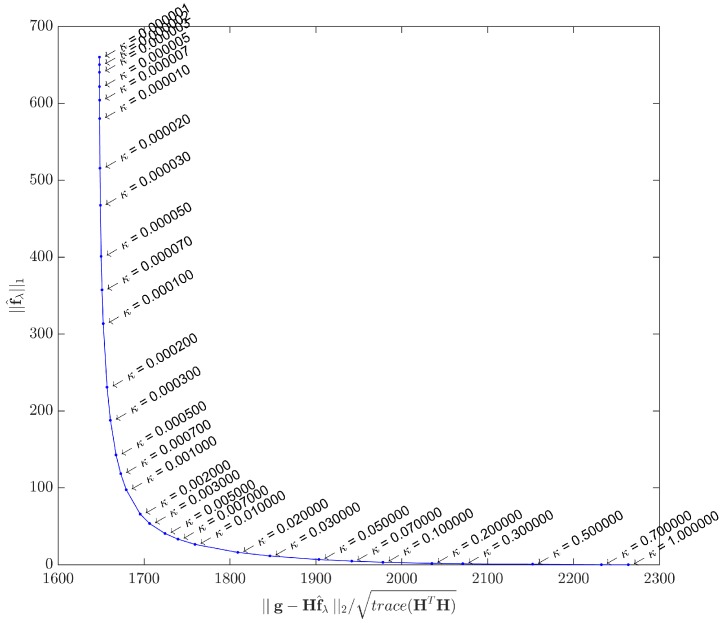
L-curve by IRLS method - Real dataset. Parameter *κ* ranges from $10^{-6}$ to 1. The curvature region is approximately delimited by $\kappa =5\times 10^{-4}$ and $\kappa =3\times 10^{-2}$.

As this study assesses the acquisition model with two distinct datasets, the L-curves were plotted for both cases. Figure 5 shows the L-curve obtained by solving Equation (1) with the synthetic dataset g, while Figure 6 shows the L-curve for the real dataset. The points in both curves correspond to solutions yielded by the IRLS method. Instead of the $log_{10}$ scale proposed in [62], we adopted different scales for the $\ell_{1}$ and $\ell_{2}$ terms in order to better highlight the corner curvatures. Although the curvature regions are clear in both cases, in Section 5 we shall use a specific metric to gain better insight about what *κ* to choose.

#### 3.6.2. NLCG Parameter *β*

Seven distinct options for this parameter were assessed, enumerated as:

1. Hager and Zhang $\beta_{HZ}$ proposed in [25];
2. Fletcher and Reeves $\beta_{FR}$ proposed in [63];
3. Dai and Yuan $\beta_{DY}$ proposed in [64];
4. Fletcher $\beta_{CD}$ proposed in [65];
5. Polak, Ribière and Polyak $\beta_{PRP}$ proposed in [66,67];
6. Hestenes and Stiefel $\beta_{HS}$ proposed in [56]; and
7. Liu and Storey $\beta_{LS}$ proposed in [68].

Total convergence times varied as much as 3.5 times among different choices, and no hybrid or parametric options were evaluated. The best convergence speeds for this study were achieved with $\beta_{HS}$.

#### 3.6.3. OMFISTA Parameters *α* and *η*

Parameter *α* has been assessed for this study with values ranging from 0.5 to 2.0, in steps of 0.1, in combination with parameter *η* ranging from 1.0 to 2.5 also in steps of 0.1. Careful evaluation of combinations revealed the best duet as being α=1.0 and η=2.0, which interestingly agrees with values for similar parameters in [69].

In the case of OMFISTA-OLS, the parameter $\alpha_{k}$ is calculated at each iteration by the line search procedure from [53]. Another set of experiments with *η* ranging from 1.0 to 2.5 has also shown its best value to be η=2.0.

#### 3.6.4. ADMM Parameter *ρ*

Given the similarity between the shrinkage-thresholding operator parameters λ/ρ and λ/c in ADMM and FISTA, respectively, we have adopted a criterion for *ρ* to be proportional to *c*. The best results for ADMM were achieved with ρ=c/4, where $c={∥H^{T}H∥}_{2}$. The same value for *c* was used in all FISTA family methods.

## 4. Materials and Methods

As mentioned, we first evaluate the discrete acquisition model represented by matrix H and the chosen algorithms with two different datasets; then, the modeling and the methods performances are further verified with a distinct dataset. For all cases, the operation parameters are summarized in Table 1. The ultrasound equipment used to collect the real dataset is supplied by Verasonics Inc., Kirkland, WA, USA (see more at: http://verasonics.com), model Vantage 128 with an L11-4v transducer. While the transducer has 128 elements, only the 64 central ones are used in order to reduce the amount of data to be handled in this study.

The reference ultrasound phantom device is called *Multipurpose Tissue/Cyst Ultrasound Phantom*, model 84-317, supplied by Fluke Corporation, Cleveland, OH, USA (see more at: http://www.flukebiomedical.com/rms/). It contains precision-spaced groups of targets in a medium that exhibits ultrasound responses similar to those found in human liver parenchyma, including the same attenuation, scattering characteristics and propagation velocity [70].

### 4.1. The Synthetic Dataset

For the case we designate as ’synthetic’, a reference 2D image is built by accurately reproducing the sizes and positions of eight small targets inside the aforementioned ultrasound phantom.

**Figure 7:**
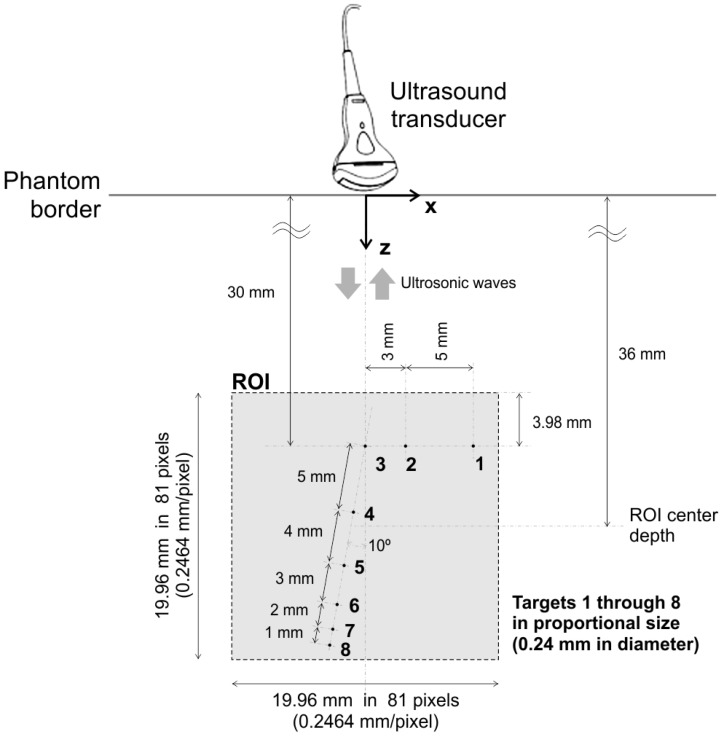
Schematic view of targets inside the phantom. Dimensions, sizes and relative positions as detailed in [70]. This picture is not present in the cited reference.

Figure 7 depicts a schematic view of the chosen targets inside the phantom, enumerated from 1 to 8. All dimensions, sizes and relative positions are exactly as described in [70]. The imaged ROI is a 19.96 mm × 19.96 mm square region, with 81×81 pixels, with a 0.2464 mm/pixel spatial resolution in both *x* and *z* axes.

**Figure 8:**
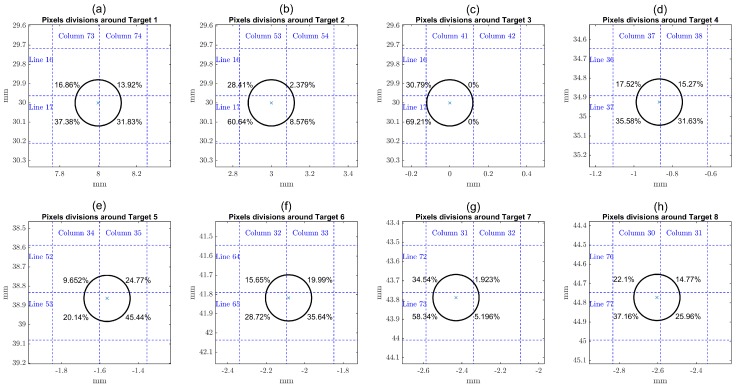
Schematic view of targets in the pixels grid.

As the real targets are round nylon rod monofilaments of 0.24 mm in diameter, and given their positions as indicated, none of them falls entirely inside a single pixel region. A schematic zoom view of the eight targets in the grid is shown in Figure 8. The percentages shown are the fractions of each target area lying inside the indicated quadrants. The reference 2D image is built by setting values in the interval [0,1] to the corresponding pixels. For example, Target 1 is represented by 4 pixels with 0.1686 and 0.1392 values in line 16, and with 0.3738 and 0.3183 values in line 17. After setting the pixels for the corresponding targets, vector $f_{synt}$ is simply obtained by stacking all 2D image columns.

**Figure 9:**
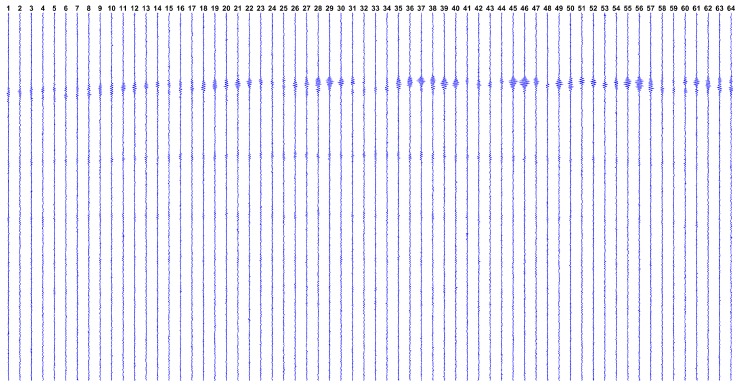
The synthetic dataset $g_{synt}=Hf_{synt}+e$. Each vertical line represents the dataset from a single transducer, over time $t_{i}$ (increasing from top to bottom).

Once $f_{synt}$ is computed, the synthetic dataset is given by $g_{synt}=Hf_{synt}+e$. The Gaussian noise vector e is calculated to produce a SNR similar to that found in the real dataset, which is estimated by:

(24) $$SNR=10\cdot log_{10}\left(\frac{{∥Hf_{synt}∥}_{2}^{2}}{{∥g_{real}∥}_{2}^{2}-{∥Hf_{synt}∥}_{2}^{2}}\right)$$

where $g_{real}$ is the real dataset collected from the ultrasound platform. The estimated SNR using this approach is -0.94 dB and the complete $g_{synt}$ dataset is depicted in Figure 9.

### 4.2. The Real Dataset

**Figure 10:**
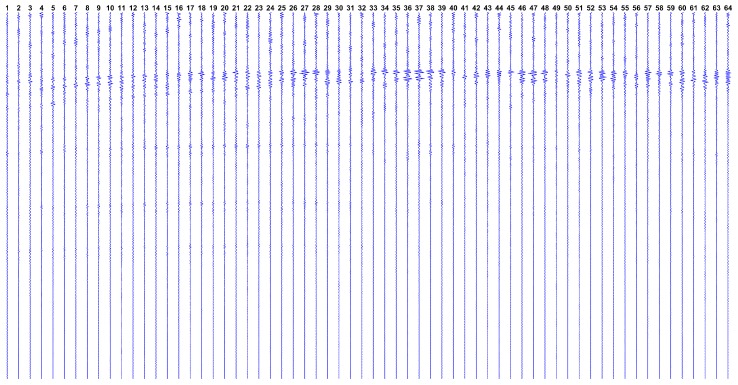
The real dataset $g_{real}$ collected by the Verasonics ultrasound research platform from Fluke phantom device. Each vertical line represents the dataset from a single transducer, over time $t_{i}$ (increasing from top to bottom).

Designated as $g_{real}$, the real dataset in collected from aforementioned ultrasound phantom, by taking the average of 10 subsequent measures. The complete $g_{real}$ dataset is shown in Figure 10.

## 5. Results and Discussion

Results for the synthetic case and the real case are presented in four different views. In addition to images, we also present results related to the algorithms performances. The same views are also presented for a distinct real dataset, in order to verify the modeling credibility.

In the first view, the resulting *reconstructed images* are presented for the 11 algorithms considered. A metric called *Array Performance Indicator* (API) proposed in [71] is used to compare the images. The API is a simple metric that quantitatively compares the performances of reconstruction methods in terms of their ability to image point-like reflectors, which is the focus of this study. The API is dimensionless and measures the size of the *Point Spread Function* (PSF). It is defined as the area ($A_{-6\mathrm{dB}}$) within which the PSF is greater than -6 dB (half amplitude) down from its maximum value, normalised to the square of the ultrasound wavelength Λ:

(25) $$API=\frac{A_{-6dB}}{\Lambda^{2}}$$

Smaller API values indicate the method has greater ability to accurately reconstruct images representing point-like targets. Larger API values mean the PSF occupies a larger area in the image, indicating less ability to image punctual objects.

The second view shows the evolution curves for the *estimation errors*, which are drawn considering the $\ell_{2}$-norm distance between an estimated solution ${\hat{f}}_{k}$ at iteration *k* and a *reference solution*. For the synthetic case, it is the image $f_{synt}$ built with the eight targets, as described in Section 4. For the real case, as the final image is unknown, we set as reference the solution $f^{\;*}$ obtained by the IRLS method after 500 iterations.

The third view presents the *cost function* Ψ(f) curves, drawn as a function of the *iteration number* *k*. These curves let us observe the efficiency of each method in reducing this functional, putting aside time considerations. In the last view, we plot the *cost function* Ψ(f) curves over *time*, in order to observe the computational cost of each method. Details are given in the following sections.

Before proceeding, however, we should specify how the value for *κ* was chosen, as it determines the regularizing parameter *λ* in Equation (23). Once the synthetic dataset was built from a *reference image* $f_{synt}$ that accurately represents the real phantom targets, we can calculate the API for this image ($API_{ref}$) and compare it with the API values ($API_{\kappa }$) of images obtained with *κ* values in the curvature region of Figure 5.

**Figure 11:**
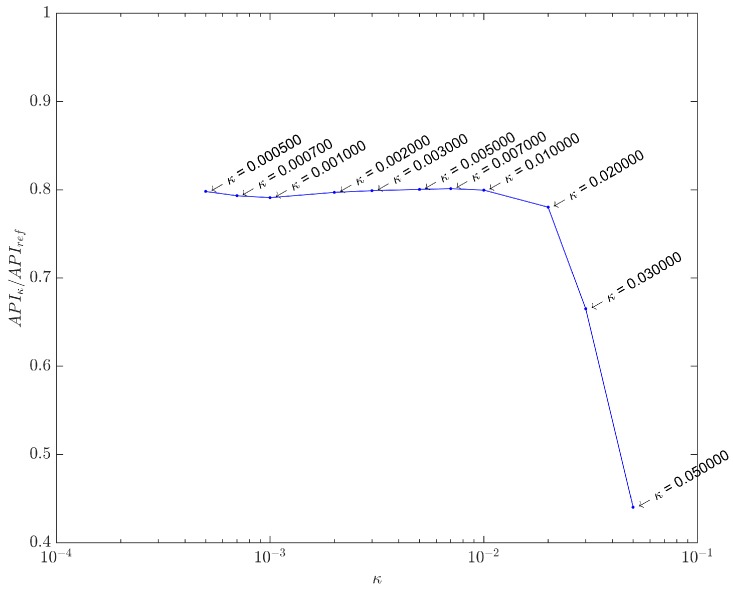
Array Performance Indicator (API) ratio curve. Solutions for each *κ* were computed with Iterative Re-weighted Least Squares (IRLS), running 30 iterations.

The ratio $API_{\kappa }/API_{ref}$ is plotted against *κ* in Figure 11, using the range $5\times 10^{-4}<\kappa <5\times 10^{-2}$ appointed in Figure 5. It is clear that beyond a certain point (κ=0.01), the ratio drops quickly, indicating an excessive sparsity has been forced. Too low ratio values indicate some important pixels carrying targets information have been suppressed, due to excessively high values for the regularizing parameter *λ*. This estimation has also been confirmed with the real dataset, showing κ=0.01 to be the best compromise in both cases. Therefore, all results presented hereafter use $\lambda =0.01\times \lambda_{max}$.

### 5.1. Results for the Synthetic Dataset

**Figure 12:**
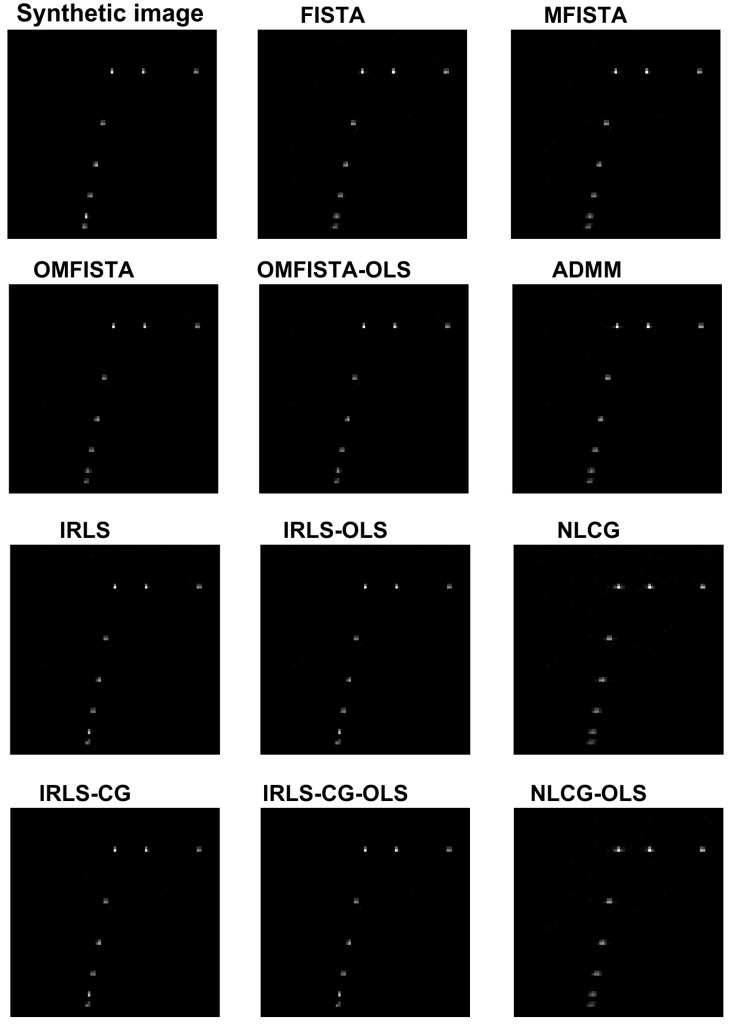
Reconstructed images from the synthetic dataset.

The reconstructed images obtained from the synthetic dataset are shown in Figure 12 and the corresponding API values are shown in Table 2. Visually, all images are very similar, but the API values and ratios point some slight differences. All IRLS family methods show similar results from the API perspective, as do NLCG methods, whose API values are slightly higher because their images are a bit more blurred than all others. Inside the FISTA group, we see a clear distinction: while FISTA and MFISTA show identical API results, OMFISTA and OMFISTA-OLS resulted in lower values. These two methods have an additional parameter to be set ($\eta_{k}$), which may have an empirical iteration-dependent update rule. In this study, this parameter was set constant, and further investigation could reveal a better approach. Despite this fact, OMFISTA-OLS has shown an API closer to the reference, partially because its stepsize is not fixed, but optimally computed at each iteration, taking the final image closer to the reference.

**Figure 13:**
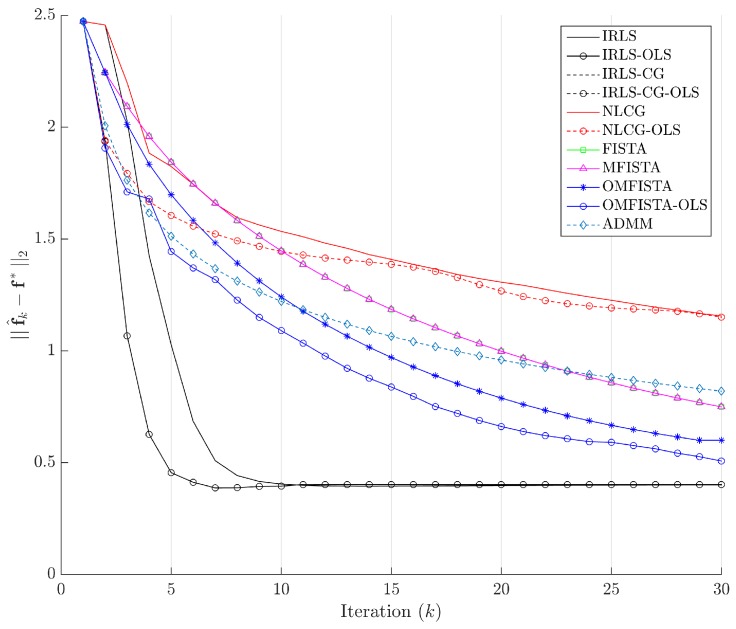
Estimation errors $||\;{\hat{f}}_{k}-f^{\;*}{\left|\right|}_{2}$ along iterations - Synthetic dataset.

The evolution curves for the estimation errors are drawn in Figure 13 for the first 30 iterations. The curves show all methods tend to converge to the same level, though some do it very slowly. Interestingly, all methods have converged to the same estimation error level after about 100 iterations, but none to zero. This means they eventually reach the same minimum region in the minimization problem, but not exactly the reference image, probably due to the high level of noise introduced in the synthetic dataset.

**Figure 14:**
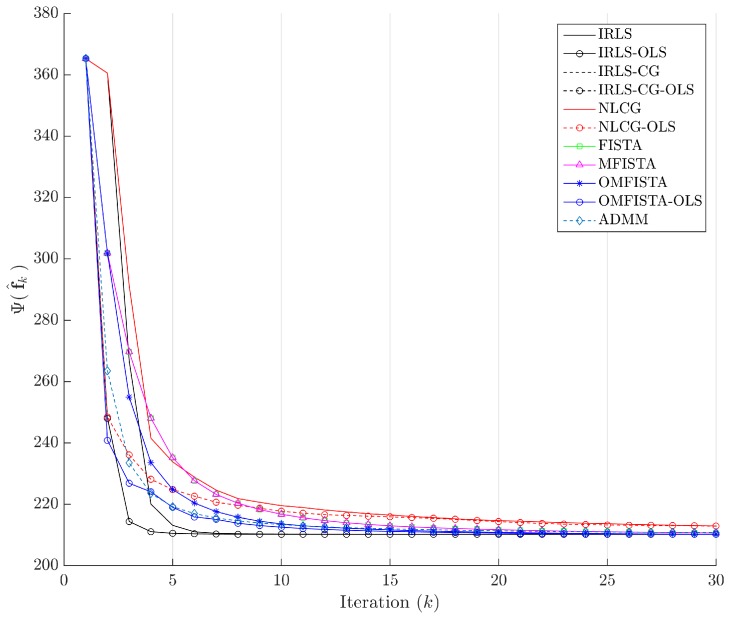
Cost function $\Psi (\;{\hat{f}}_{k}\;)$ versus iteration number *k*—Synthetic dataset.

**Figure 15:**
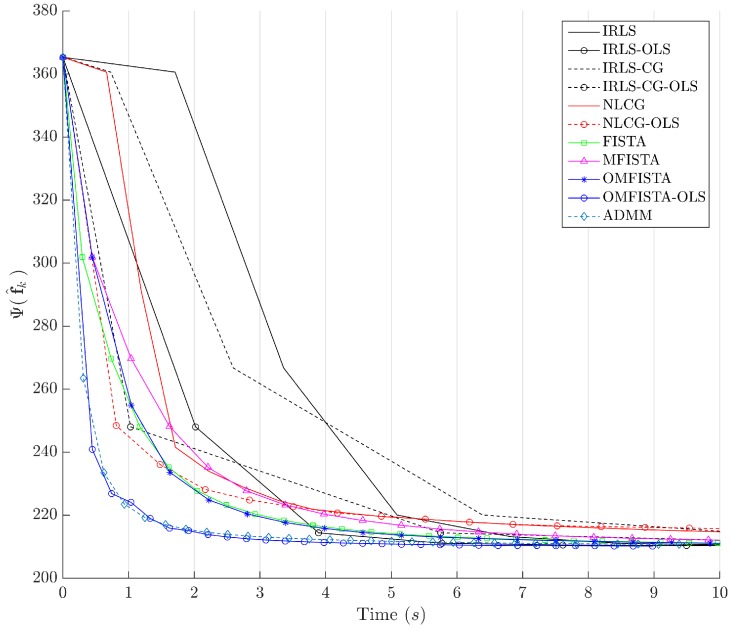
Cost function $\Psi (\;{\hat{f}}_{k}\;)$ versus time in seconds—Synthetic dataset.

The cost function curves are drawn against the iteration number *k* and against time, in Figure 14 and Figure 15, respectively. The former indicate all methods eventually converge to the same cost level, but do take different numbers of steps to reach the minimum. The latter is presented with a closer look at the initial computation instants; all time measurements refer to algorithms running on an Intel^®^ Core^™^ i7-4790 CPU (Intel Corporation, Santa Clara, CA, USA) at 3.60 GHz, with 32 GB DDR3 RAM, over Microsoft Windows 7 Professional© 64 bits, running MATLAB^®^ (The MathWorks Inc., Natick, MA, USA).

It is clear that some methods are much more time-efficient than others. The acceleration promoted by the line search procedure for OLS-suffixed algorithms becomes evident when they are compared to their non-OLS counterparts. Time efficiency is also manifest when all methods are grouped in the aforementioned families division. NLCG and IRLS-derived methods are the most time consuming. FISTA methods are well suited for this type of problem, with clear advantage for OMFISTA-OLS, which presents almost the same convergence speed as ADMM.

### 5.2. Results for the Real Dataset

While the synthetic dataset is well known and controlled, we should notice some particularities of the real dataset. Besides sampling errors and other noise signals inherent to sampled data from real transducers, we remark two other sources of slight variation between the synthetic and the real cases.

First, every real device present dimensions and material properties within a given variance range (detailed information about the phantom device may be found at: http://www.flukebiomedical.com/biomedical/usen/diagnostic-imaging-qa/ultrasound-qa/84-317-multi-purpose-tissue-cyst-ultrasound-phantom.htm?PID=55394). For instance, the real speed of sound may vary as much as ±6 m/s in the phantom material, while stated positions and diameters of targets may vary as much as ±0.10 mm and ±5%, respectively.

Second, the distance, inclination and translation of the ultrasound transducers relative to targets may be slightly different from the ones indicated in Figure 7, due to the use of the coupling gel, and due to the inability and/or inaccuracy of the machine operator while scanning.

**Figure 16:**
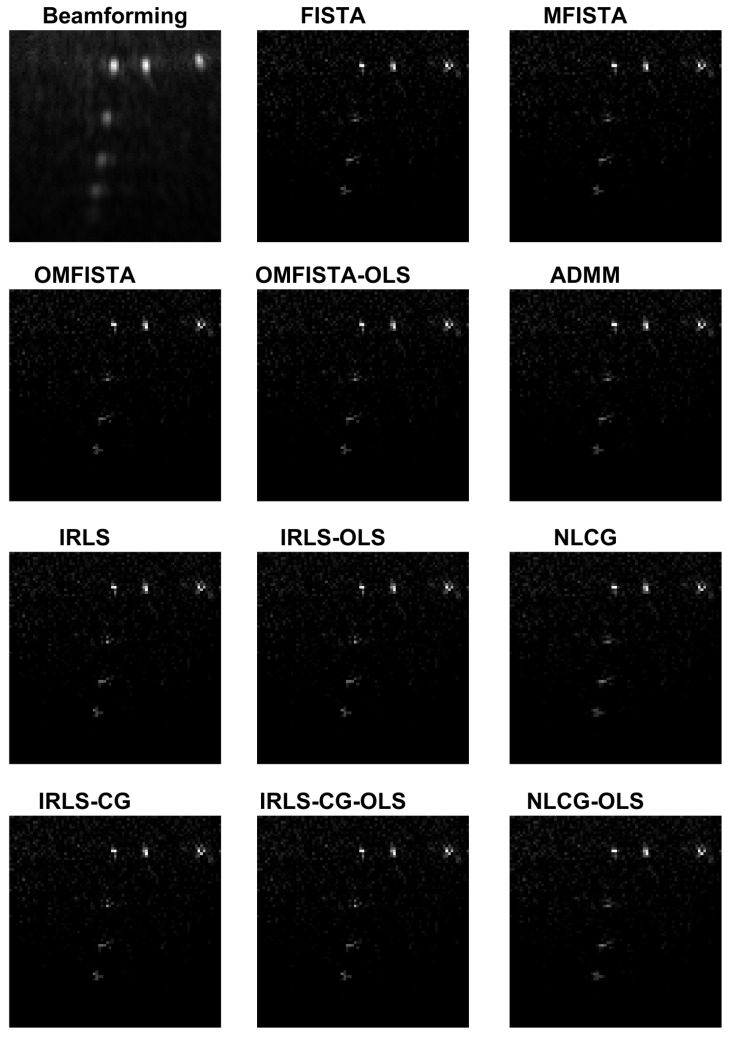
Reconstructed images from the real dataset.

The reconstructed images obtained from the real dataset are shown in Figure 16 and the corresponding API values are shown in Table 3. Again, all images are visually very similar, and the API values and ratios are used to highlight the differences.

From the API perspective, the conclusion for IRLS, NLCG and FISTA families are generally the same as stated for the synthetic case, except here the API ratios are more concise, all close to unity. The clear exception is the API measure for the beamforming image, which clearly indicates this traditional imaging method has limited capability of representing the targets diameters in real proportion to the image. This result confirms our claim that iterative methods may yield more precise results if care is taken to build a proper acquisition model.

**Figure 17:**
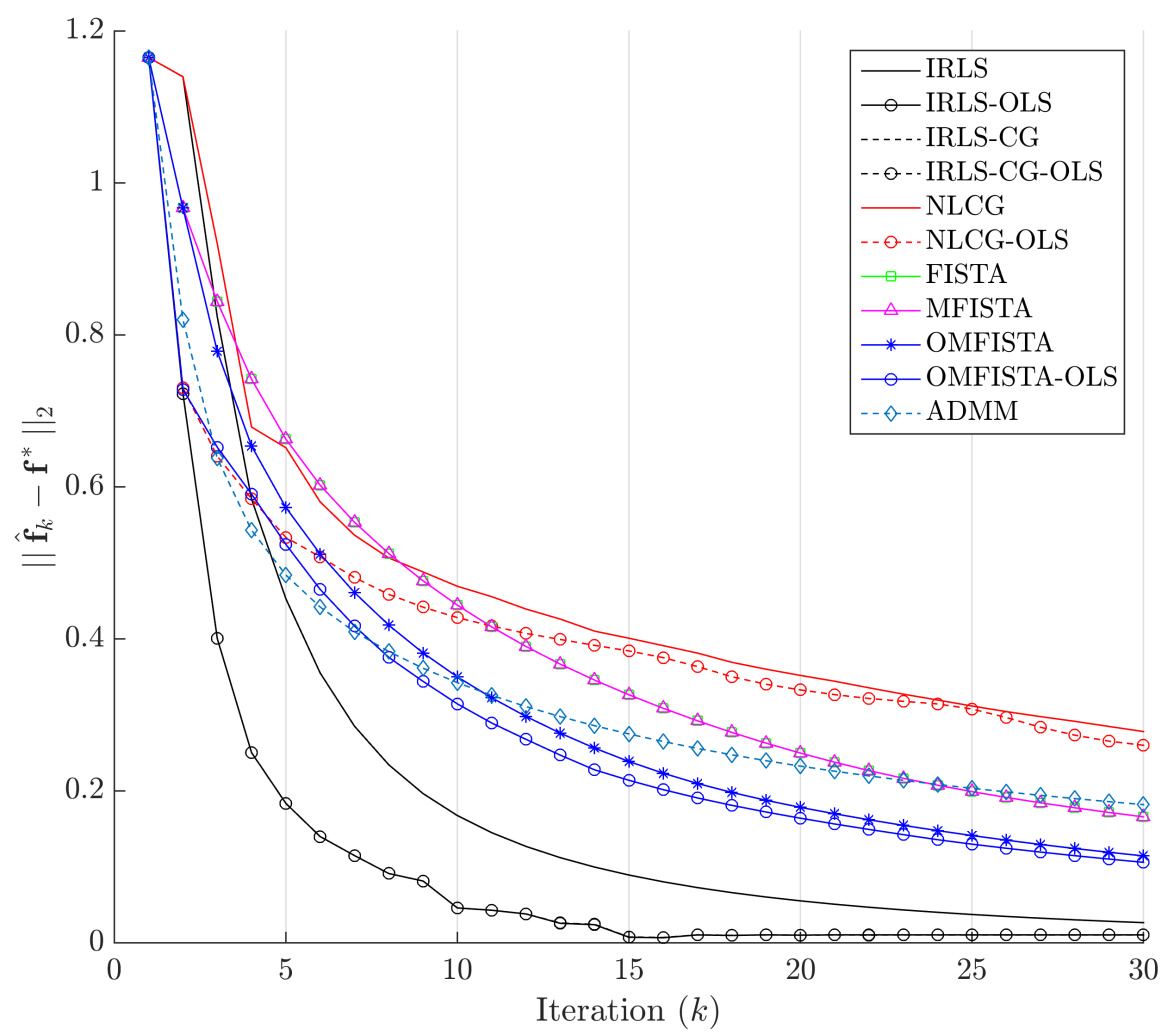
Estimation errors $||\;{\hat{f}}_{k}-f^{\;*}{\left|\right|}_{2}$ along iterations—Real dataset.

The evolution curves for the estimation errors are drawn in Figure 17 for the first 30 iterations. Here again, the curves show all methods tend to converge to the same level, but now all errors tend to zero, meaning all considered algorithms tend to reach the reference solution $f^{\;*}$, calculated by IRLS with 500 iterations.

**Figure 18:**
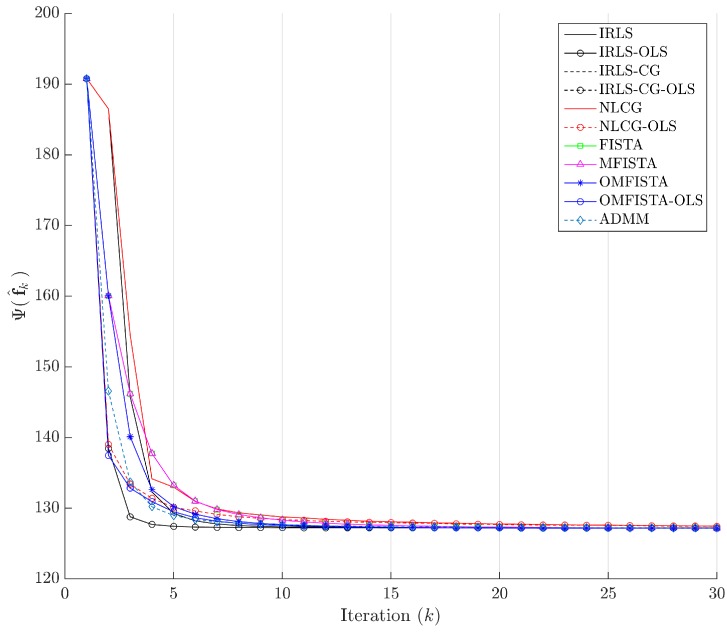
Cost function $\Psi (\;{\hat{f}}_{k}\;)$ versus iteration number *k*—Real dataset.

**Figure 19:**
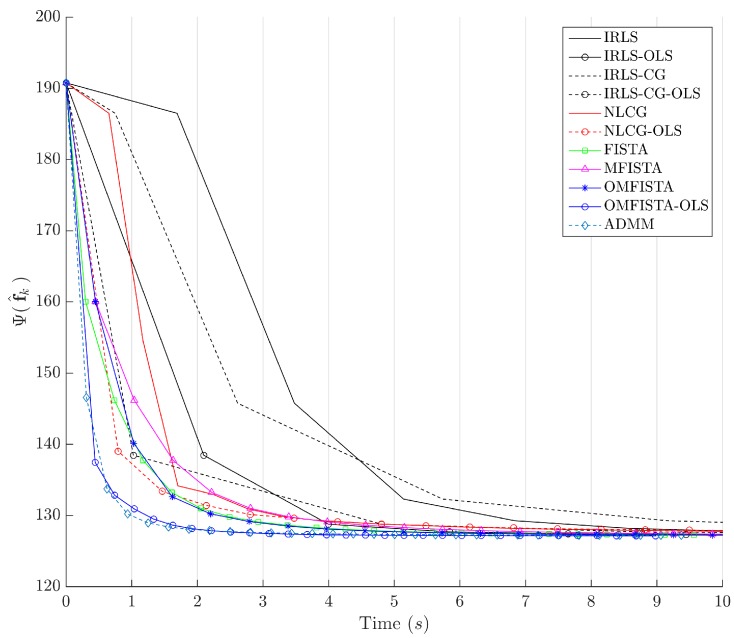
Cost function $\Psi (\;{\hat{f}}_{k}\;)$ versus time in seconds—Real dataset.

The cost function curves are drawn against the iteration number *k* and against time, in Figure 18 and Figure 19, respectively. As before, the former indicates all methods converge to the same cost level, with different numbers of steps, while the latter highlights distinct computational costs; all time measurement were taken in the same computing environment described before.

### 5.3. Results for a Distinct Real Dataset

In order to verify the accuracy of the proposed modeling procedure, the algorithms are now used to reconstruct images from a distinct real dataset. The data are obtained with the same ultrasound equipment, but the four targets rely in a different region of the same phantom previously used. Matrix H is built for a proper ROI, encompassing the new targets, and consists of a 17.99 mm × 21.93 mm rectangular region, with 73×89 pixels, with a 0.2464 mm/pixel spatial resolution in both *x* and *z* axes. No information about the ’real’ image is available, except for the targets nature, which consist of nylon rod monofilaments of 0.24 mm in diameter as before.

In terms of parameters, the same values for *λ*, *β*, *α* and *η* have been used. The parameter $c={∥H^{T}H∥}_{2}$ has been computed for the new model H, and ρ=c/4 as before.

**Figure 20:**
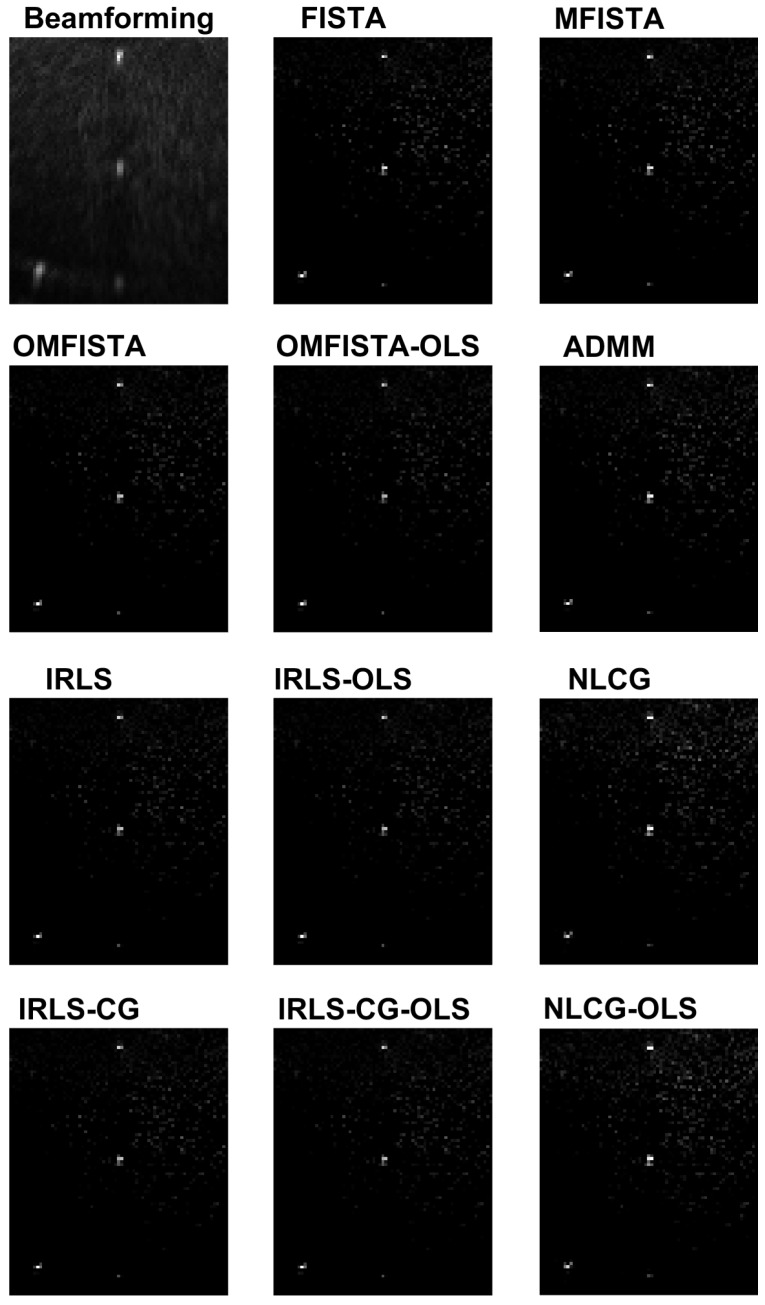
Reconstructed images from a distinct real dataset, of a different ROI.

The reconstructed images obtained by all methods are shown in Figure 20 and the corresponding API values are shown in Table 4. Again, all images are visually very similar, and the API values and ratios are used to highlight the differences.

From the API perspective, we notice that all IRLS methods yield very similar results. The FISTA family is also consistent, with the lower API ratio given by the OMFISTA-OLS algorithm. NLCG methods and ADMM have resulted in a bit more blurred images, which explains their higher API ratios. This is confirmed by the estimation error curves below, where these algorithms present slower convergence. Even so, as before, the beamforming method has shown the poorest API result.

**Figure 21:**
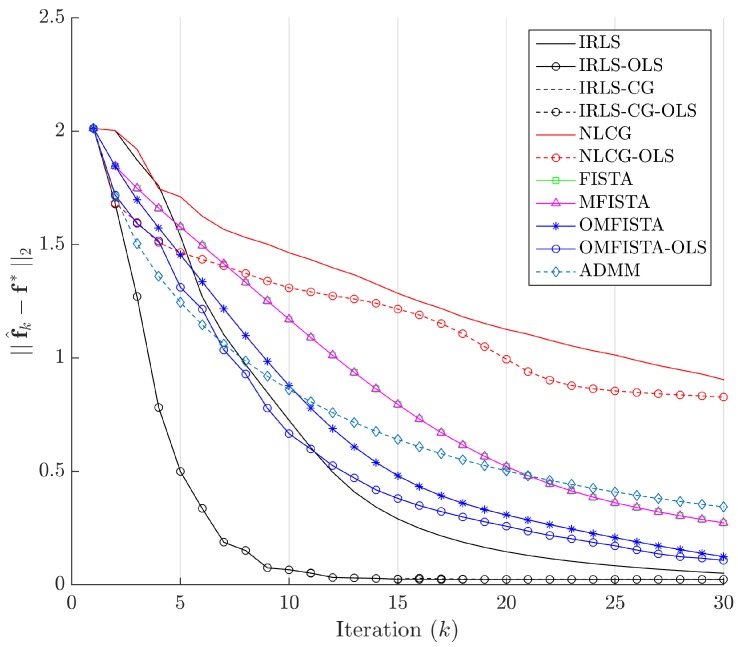
Estimation errors $||\;{\hat{f}}_{k}-f^{\;*}{\left|\right|}_{2}$ along iterations—Distinct real dataset.

The evolution curves for the estimation errors are shown in Figure 21 for the first 30 iterations. As mentioned, some methods show slower convergence, but the behaviour within and among families is the same as before.

**Figure 22:**
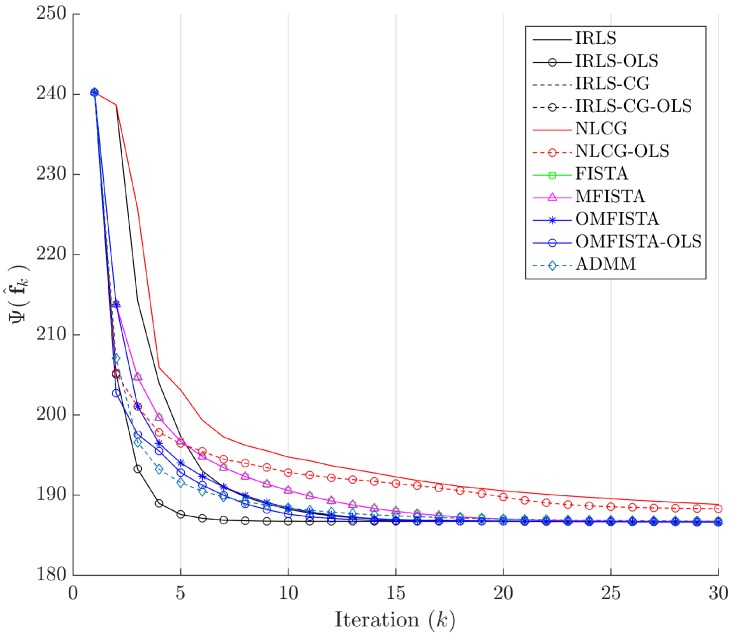
Cost function $\Psi (\;{\hat{f}}_{k}\;)$ versus iteration number *k*—Distinct real dataset.

**Figure 23:**
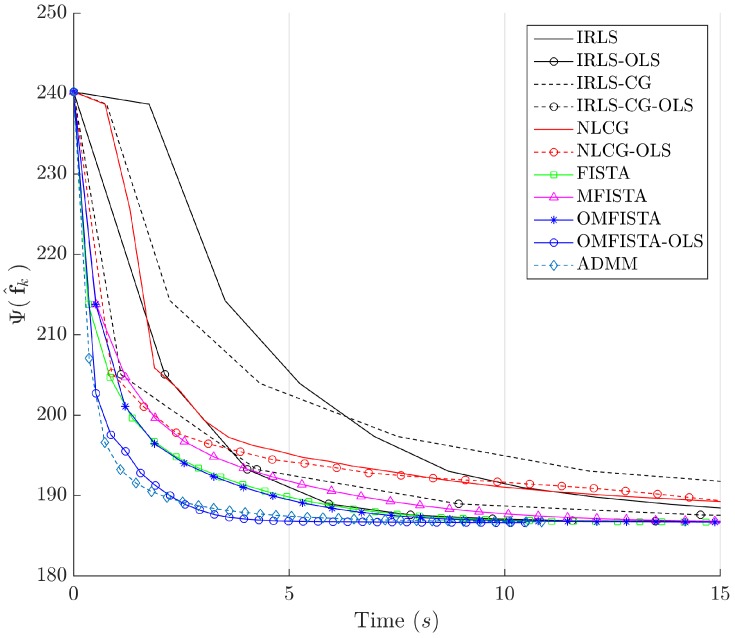
Cost function $\Psi (\;{\hat{f}}_{k}\;)$ versus time in seconds—Distinct real dataset.

Finally, the cost function curves are shown against the iteration number *k* and time, in Figure 22 and Figure 23, respectively. As before, the former indicates all methods converge to the same cost level, but now the convergence speeds differences are more noticeable. The latter highlights distinct computational costs, with all time measurements taken as described before.

### 5.4. A Note about the Stop Criterion

We should remark the reason why we have chosen a fixed number of k=30 iterations as the stop criterion for the iterative algorithms.

**Figure 24:**
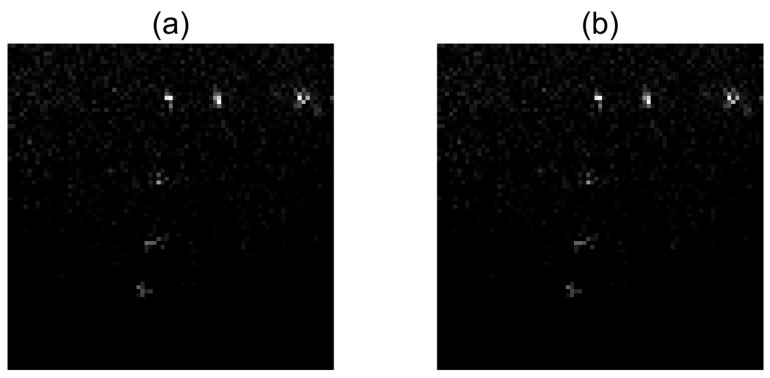
Real dataset—(**a**) Reference image $f^{\;*}$ obtained by IRLS after 500 iterations; (**b**) Image ${\hat{f}}_{k}$ obtained by IRLS after 30 iterations. API values are given in Table 3.

**Figure 25:**
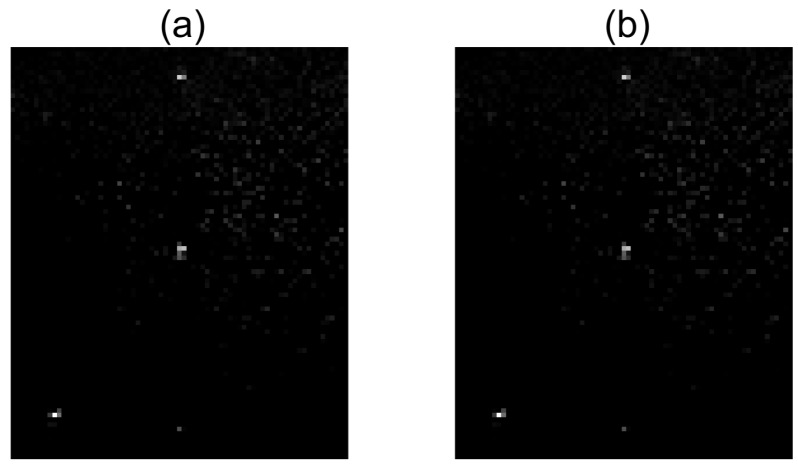
Distinct real dataset—(**a**) Reference image $f^{\;*}$ obtained by IRLS after 500 iterations; (**b**) Image ${\hat{f}}_{k}$ obtained by IRLS after 30 iterations. API values are given in Table 4.

If we observe the curves of estimation errors $||\;{\hat{f}}_{k}-f^{\;*}{\left|\right|}_{2}$ for IRLS in Figure 17 and Figure 21, we notice their final values represent approximately 2.3% and 2.5% of the initial errors, respectively. These low percentages explain why the images obtained by IRLS after 30 iterations are visually so close to the reference images $f^{\;*}$ achieved by the same method after 500 iterations, as shown in Figure 24 and Figure 25.

In practice, however, iterative algorithms are often used with a stop criterion defined in terms of the variation of the cost function, since in image reconstruction we seldom have a reference image available to compute the estimation error. The algorithm usually stops when the cost function does not decrease beyond a certain amount between two consecutive iterations. In Figure 18 and Figure 22 we see this general trend for k>25, and therefore we have chosen k=30 as the stop criterion for all algorithms in this study.

## 6. Conclusions

Although limited, the proposed acquisition model has shown satisfactory results, both visually and by inspection of the API metric. The strategy for the model evaluation, by first elaborating a synthetic dataset with a reference image that mimics the real phantom device, has allowed us to better adjust some parameters in a controlled situation, before assessing the model with the real dataset. A distinct dataset with real data collected from different targets have shown the modeling to be reasonable, as all methods have reached good results.

As claimed, although high computational effort is required by iterative algorithms, solving the posed $\ell_{2}$–$\ell_{1}$ problem with the proposed discrete model may lead to images closer to ground-truth than traditional beamforming imaging. In this study, although all considered algorithms have been able to show good reconstructed images, the computational performances have varied considerably. The best performances have been observed with OMFISTA-OLS and ADMM methods, and further acceleration of the latter is being investigated by the authors. Nonetheless, computing capabilities of current platforms need to evolve before frame rates currently delivered by ultrasound equipments are achievable.

As mentioned, the discrete acquisition model is limited by the inherent errors from both time and space sampling, and further investigation on the topic is being carried within the authors research team. Some alternative approaches are under evaluation, such as using a complex-valued model or using an online function to calculate H elements as iterations proceed, in order to optimize memory requirements for computation. In this study, while the computer used for the calculations has a multi-core processor, all MATLAB^®^ routines were written for sequential processing. As some calculations may also be carried in parallel, this may reveal a good source of reconstruction acceleration, and future papers will address this issue.

## Figures and Tables

**Table 1:** Main parameters used in the ultrasonic imaging system.

Parameter	Specification
Ultrasound frequency $\left(f_{c}\right)$	6.25 MHz
Sampling frequency $\left(f_{S}\right)$	25 MHz
Speed of sound (v)	1540 m/s
Attenuation rate (liver parenchyma)	0.5 dB/cm/MHz
Wavelength (Λ)	0.2464 mm
Number of transducer elements (array)	128
Transducer bandwidth (B)	3.84 MHz
Fractional bandwidth (BW)	61.44%

**Table 2:** API values for reconstructed images after 30 iterations—Synthetic dataset.

**Reference Image**	**${\mathrm{API}}_{ref}$**	
Synthetic phantom image	5.4813	
**Reconstruction Method**	API	$\mathrm{API}/{\mathrm{API}}_{ref}$
IRLS	4.4000	0.80
IRLS-OLS	4.4029	0.80
IRLS-CG	4.4000	0.80
IRLS-CG-OLS	4.4029	0.80
NLCG	6.1278	1.12
NLCG-OLS	6.1292	1.12
FISTA	5.7841	1.06
MFISTA	5.7841	1.06
OMFISTA	3.4132	0.62
OMFISTA-OLS	4.0858	0.75
ADMM	5.8548	1.07

**Table 3:** API values for reconstructed images after 30 iterations - Real dataset .

**Reference image**	**${\mathrm{API}}_{ref}$**	
IRLS after 500 iterations	7.2347	
**Reconstruction Method**	API	$\mathrm{API}/{\mathrm{API}}_{ref}$
Beamforming	20.8626	2.88
IRLS	7.2194	1.00
IRLS-OLS	7.2307	1.00
IRLS-CG	7.2194	1.00
IRLS-CG-OLS	7.2303	1.00
NLCG	6.9467	0.96
NLCG-OLS	6.9748	0.96
FISTA	7.4016	1.02
MFISTA	7.4016	1.02
OMFISTA	6.5906	0.91
OMFISTA-OLS	6.5703	0.91
ADMM	6.9921	0.97

**Table 4:** API values for reconstructed images after 30 iterations—Distinct real dataset.

**Reference Image**	**${\mathrm{API}}_{ref}$**	
IRLS after 500 iterations	3.7327	
**Reconstruction method**	API	$\mathrm{API}/{\mathrm{API}}_{ref}$
Beamforming	5.3886	1.44
IRLS	3.7378	1.00
IRLS-OLS	3.7312	1.00
IRLS-CG	3.7378	1.00
IRLS-CG-OLS	3.7298	1.00
NLCG	4.8393	1.30
NLCG-OLS	4.6477	1.25
FISTA	4.3360	1.16
MFISTA	4.3360	1.16
OMFISTA	3.8639	1.04
OMFISTA-OLS	3.1716	0.85
ADMM	4.6643	1.25

## Abbreviations

The following abbreviations are used in this manuscript:

ADMM	Alternating Direction Method of Multipliers
API	Array Performance Indicator
FISTA	Fast Iterative Shrinkage-Thresholding Algorithm
IPB	Inverse Problem-Based approach
IRLS	Iteratively Re-weighted Least Squares
IRLS-OLS	Iteratively Re-weighted Least Squares with Optimal Line Search
IRLS-CG	Iteratively Re-weighted Least Squares with Conjugate Gradient
IRLS-CG-OLS	Iteratively Re-weighted Least Squares with Conjugate Gradient and Optimal Line Search
ISTA	Iterative Shrinkage-Thresholding Algorithm
MFISTA	Monotone Fast Iterative Shrinkage-Thresholding Algorithm
NLCG	Nonlinear Conjugate Gradient
NLCG-OLS	Nonlinear Conjugate Gradient with Optimal Line Search
OMFISTA	Over-Relaxation of Monotone Fast Iterative Shrinkage-Thresholding Algorithm
OMFISTA-OLS	Over-Relaxation of Monotone Fast Iterative Shrinkage-Thresholding Algorithm with
	Optimal Line Search
PSF	Point Spread Function
ROI	Region Of Interest
SNR	Signal-to-Noise Ratio
